# Malaria Test Positivity, Temporal Trends, and Associated Factors Among Clinically Suspected Adult Cases in Maruleng Sub-District, Limpopo Province, South Africa, 2018–2023

**DOI:** 10.3390/ijerph23070866

**Published:** 2026-07-02

**Authors:** Stella Mashego, Tanusha Singh

**Affiliations:** Department of Environmental Health, University of Johannesburg, Johannesburg 2028, South Africa; smashego6@gmail.com

**Keywords:** malaria, test positivity, seasonal trends, socioeconomic risk factors, behavioural risk factors, environmental risk factors

## Abstract

**Highlights:**

**Public health relevance—How does this work relate to a public health issue?**

- This study provides six years of sub-district-level evidence on malaria test positivity among clinically suspected adults attending public healthcare facilities in the Maruleng Sub-District of Limpopo Province.
- It provides information on who is most affected by malaria, when malaria cases are most common, and how malaria patterns change over time in a rural setting with limited local evidence.

**Public health significance—Why is this work of significance to public health?**

- The findings show that malaria remains a significant concern among adults seeking care, particularly young adult males.
- The study demonstrates how routine surveillance data can be used to identify local trends, seasonal peaks, and areas with a higher positivity rate of malaria, to guide control efforts.

**Public health implications—What are the key implications or messages for practitioners, policy makers and/or researchers in public health?**

- Malaria prevention and control activities should be strengthened in high-positivity areas such as Hoedspruit, with a focus on early detection, prompt treatment, and mosquito control measures.
- Improving the quality and completeness of malaria surveillance data will support better decision-making and help health authorities respond more effectively to malaria risks.

**Abstract:**

Background: Malaria remains a significant public health challenge in sub-Saharan Africa and is endemic in certain areas of South Africa despite ongoing elimination efforts. This study assessed malaria positivity, temporal trends, seasonal patterns, and factors associated with malaria positivity among clinically suspected adult in Maruleng Sub-District, Limpopo Province. Methods: A retrospective cross-sectional study analysed routine malaria surveillance records from 2018 to 2023 among suspected adult cases (≥18 years) tested for malaria at 11 clinics and one hospital. Descriptive, regression, and seasonal trend analyses were performed. Results: Of 385 adult records analysed, 366 were malaria-positive (test positivity rate = 95.1%). Cases were mostly males (66.7%) and adults aged 18–35 years (47.5%). Malaria cases were highest in 2018 and 2019, declined between 2020 and 2022, and increased in 2023. Significant seasonal variation was observed, with the highest numbers of cases occurring in April and May (*p* < 0.001). Exploratory regression analyses did not identify any statistically significant independent predictors of malaria positivity after adjustment for potential confounders. Behavioural risk factors showed a non-significant trend towards increased odds of malaria positivity (aOR = 3.42; 95% CI: 0.78–14.98), although estimates should be interpreted cautiously because of the limited number of malaria-negative records. Conclusions: Malaria remains an important public health concern in Maruleng. The observed temporal and seasonal patterns highlight the importance of strengthening surveillance, seasonal preparedness, and targeted vector control interventions before peak transmission periods. Larger studies with greater outcome variability are needed to better characterise factors associated with malaria positivity.

## 1. Introduction

Malaria remains one of the most significant vector-borne diseases (VBDs) worldwide and continues to pose a major public health challenge, particularly in low- and middle-income countries (LMICs) [1]. According to the World Health Organization (WHO), an estimated 263 million malaria cases and 597,000 deaths occurred worldwide in 2023, with approximately 95% of malaria-related deaths occurring in the WHO African Region [2]. Nevertheless, malaria control interventions prevented more than 170 million cases and one million deaths globally in 2024, mostly from the WHO African Region [3].

The WHO Global Technical Strategy for Malaria 2016–2030 aims to reduce malaria incidence and mortality by 90% by 2030, eliminate malaria in at least 35 countries, and prevent its re-establishment in malaria-free areas [4]. Despite these initiatives, malaria remains a substantial burden in sub-Saharan Africa, where environmental, climatic, socioeconomic, and health-system factors sustain transmission [5,6].

South Africa has made considerable progress in reducing malaria transmission in recent decades; however, the disease remains endemic in certain areas, particularly in the northeastern provinces of Limpopo, Mpumalanga, and KwaZulu-Natal [7,8]. Transmission is largely seasonal and typically occurring during the rainy months between September and May when environmental conditions favour mosquito breeding [7]. Cross-border migration from neighbouring malaria-endemic countries such as Mozambique, Zimbabwe, Malawi, and Eswatini further contributes to imported infections and complicates elimination efforts [9]. South Africa reported approximately 28,000 malaria cases in 2017, with Limpopo Province accounting for most cases and recording the highest incidence rate (331 cases per 100,000 population) [10,11]. Between 2015 and 2017, 43,199 malaria cases were reported nationally, of which 3.5% were imported [11]. By 2019, reported cases had declined to approximately 7900, with most occurring in Limpopo, Mpumalanga, and KwaZulu-Natal provinces [12].

The Maruleng Sub-District, located in Mopani District Municipality in Limpopo Province, continues to experience seasonal malaria outbreaks. Despite ongoing control measures, including indoor residual spraying, improved diagnostics, and surveillance programmes, malaria continues to affect residents in the area [13]. However, limited published evidence exists on malaria patterns among adults at the sub-district level, particularly regarding temporal trends and demographic risk factors. Most South African malaria studies report findings at provincial or district level, with little information available on facility-based malaria positivity, seasonal variation, and population characteristics within Maruleng Sub-District. This evidence gap limits the ability of local programme managers to identify high-risk groups, target interventions, and allocate resources effectively. Understanding local malaria patterns is essential for guiding targeted control measures and supporting malaria elimination efforts. This study assessed malaria test positivity, temporal trends, seasonal patterns, and factors associated with malaria positivity among suspected adult cases attending public health facilities in Maruleng Sub-District, Limpopo Province, using routine surveillance data collected between 2018 and 2023.

## 2. Materials and Methods

### 2.1. Study Design

A retrospective observational study was conducted using routine malaria surveillance data. Cross-sectional analyses assessed factors associated with malaria positivity, while descriptive analyses examined annual and seasonal trends between 2018 and 2023.

### 2.2. Study Setting

The study was conducted in Maruleng Sub-District, Mopani District Municipality, Limpopo Province, South Africa. The predominantly rural area comprises 12 wards and 23 villages, including farms, tourism facilities, private nature reserves, and areas bordering the Kruger National Park. Malaria surveillance is coordinated through the Maruleng Malaria Control Centre (MMCC), which compiles data from 11 clinics and one regional hospital serving the sub-district. Environmental factors such as seasonal rainfall, irrigation practices, dams, vegetation cover, and stagnant water bodies provide favourable conditions for mosquito breeding. In addition, occupational activities associated with farming, tourism, and outdoor labour may increase exposure to mosquito bites.

### 2.3. Study Population

The study included all adults aged 18 years and older with malaria test records captured in the MMCC surveillance database between 1 January 2018 and 31 December 2023. The study population therefore comprised adults tested for malaria within the surveillance system rather than the general adult population of Maruleng Sub-District. The study was restricted to public-sector surveillance records because private-sector data were not available in the MMCC database.

#### 2.3.1. Inclusion Criteria

Adults aged 18 years and older with documented malaria test results in the MMCC database between 1 January 2018 and 31 December 2023 were included. Adults were specifically selected because malaria risk factors, exposure patterns, and clinical characteristics differ from those of children. Focusing on adults allowed for a more targeted assessment of malaria positivity and associated factors in this population. All eligible records were included, and no sampling was undertaken.

#### 2.3.2. Exclusion Criteria

Individuals younger than 18 years, records from private healthcare providers, and records with missing malaria test outcomes were excluded. The analysis was limited to routinely collected public-sector surveillance data because private-sector records were not available in the study database. As healthcare utilisation may differ between the public and private sectors, the findings should be interpreted within the context of the public healthcare system.

### 2.4. Variables

The primary outcome variable was malaria test status, classified as positive or negative based on the recorded diagnosis. Variables extracted from the surveillance database included age, sex, race, nationality, employment status, tracing status, previous malaria infection, travel history, clinical outcome, and environmental, behavioural, and socioeconomic risk factors. Environmental risk factors included conditions favourable for malaria transmission, such as irrigation, vegetation, dams, open water bodies, poor drainage, rainfall, humidity, and high temperatures. Behavioural risk factors included activities that may increase mosquito exposure, such as outdoor night-time activities and inconsistent use of repellents, insecticide-treated nets, or protective clothing. Socioeconomic risk factors included agricultural work, unemployment, poverty, inadequate housing, and low educational attainment. These variables were as routine operational classifications during malaria case investigations and were captured in the surveillance database as binary variables (Yes/No). The dataset did not contain detailed information regarding the specific criteria or scoring process used to assign these classifications, and therefore the variables were analysed according to the recorded surveillance classifications. For regression analyses, malaria test status was modelled as a binary outcome (positive = 1, negative = 0). Explanatory variables included age group, sex, nationality, tracing status, previous malaria infection, socioeconomic risk factors, behavioural risk factors, and travel history. Clinical outcome (deceased) was excluded from the regression models because it occurs after diagnosis and is not a determinant of malaria positivity.

### 2.5. Data Sources and Measurement

Data were extracted from the malaria surveillance register maintained by the MMCC. A structured extraction sheet (Table S1) was used to collect relevant variables from the database. Malaria testing at primary healthcare clinics was primarily performed using Rapid Diagnostic Tests (RDTs) (Abbott Diagnostics, Yongin-si, Republic of Korea) as part of routine case management. In selected cases, patients were referred for further investigation and microscopy confirmation at hospital level. The final malaria test status was determined using the diagnosis in the surveillance database. Records indicating a negative confirmatory blood smear result were classified as malaria-negative, thereby reflecting the final recorded malaria status rather than the initial screening result alone. Data collection dates were additionally used to assess monthly malaria case distributions and seasonal variation over the study period.

### 2.6. Bias

As this study used routinely collected surveillance data, there was potential for incomplete recording, missing information, and misclassification of selected variables. These limitations were considered during the interpretation of the findings. Records with missing malaria test outcomes were excluded from the analysis in accordance with the study exclusion criteria.

### 2.7. Study Size

A total of 385 eligible adult records were reviewed. This represented all records that met the inclusion criteria during the study period.

### 2.8. Statistical Methods

Statistical analyses were performed using IBM SPSS Statistics version 30 (IBM Corp., Armonk, NY, USA), with data management conducted in Microsoft Excel (Microsoft Corporation, Redmond, WA, USA). Descriptive statistics were used to summarise demographic characteristics, facility distribution, malaria positivity, and temporal trends. Continuous variables were described using means and standard deviations (SD), while categorical variables were summarised using frequencies and percentages. Malaria test positivity was calculated as the proportion of positive cases among all adults tested. Bivariate analyses compared malaria-positive and malaria-negative records using Pearson’s chi-square (χ^2^) or Fisher’s exact tests, as appropriate. Logistic regression was used to investigate factors associated with malaria positivity, with malaria test status modelled as a binary outcome (positive = 1, negative = 0). Incidence rates could not be calculated because population denominator data were unavailable. Variables with a bivariate association of *p* < 0.20 were considered for inclusion in the multivariable model. Age group and sex were retained regardless of statistical significance because they were considered potential confounders, while travel history and behavioural risk factors were retained based on their established biological relevance to malaria transmission. Crude odds ratios (ORs), adjusted odds ratios (aORs), 95% confidence intervals (CIs), and *p*-values were reported, with statistical significance set at *p* < 0.05. Temporal trends were assessed using annual case counts, while seasonal variation was examined by aggregating monthly malaria-positive cases across the study period. A χ^2^ goodness-of-fit test was used to assess whether cases were evenly distributed across months. Multicollinearity among exploratory variables was assessed using variance inflation factors (VIFs), with VIF values < 5 indicating no evidence of problematic collinearity. Given the small number of malaria-negative records available for comparison (*n* = 19), the logistic regression analyses were considered exploratory and interpreted cautiously. The highly imbalanced outcome distribution may have reduced statistical power and contributed to unstable parameter estimates and wide confidence intervals.

## 3. Results

### 3.1. Sociodemographic and Clinical Characteristics of Malaria Cases

Malaria testing was primarily conducted among clinically suspected cases attending public healthcare facilities. Therefore, the findings reflect malaria positivity and associated characteristics among tested adults captured within the surveillance system rather than malaria risk in the general adult population of Maruleng Sub-District. Table 1 presents the sociodemographic and clinical characteristics of adults tested for malaria in Maruleng Sub-District between 2018 and 2023. A total of 385 adult malaria test records were reviewed, of which 366 (95.1%) were malaria-positive and 19 (4.9%) were malaria-negative (Table 1). The mean age was 38.1 years, 38.3 years among malaria-positive adults, and 34.2 years among malaria-negative adults. Males constituted 67.3% of the total sample and the largest age group was 18–35 years (48.3%). Age group and sex did not differ significantly by malaria test status. Most participants were black (93.8%), South African nationals accounted for (86.2%) and most malaria-positive adults (79.0%) reported no travel history outside South Africa. Significant differences between malaria-positive and malaria-negative records were observed for nationality (*p* = 0.003), socioeconomic risk factors (*p* = 0.030), and travel history (*p* = 0.031), while employment status showed borderline statistical significance (*p* = 0.050).

### 3.2. Facility Distribution of Malaria Cases

**Figure 1:**
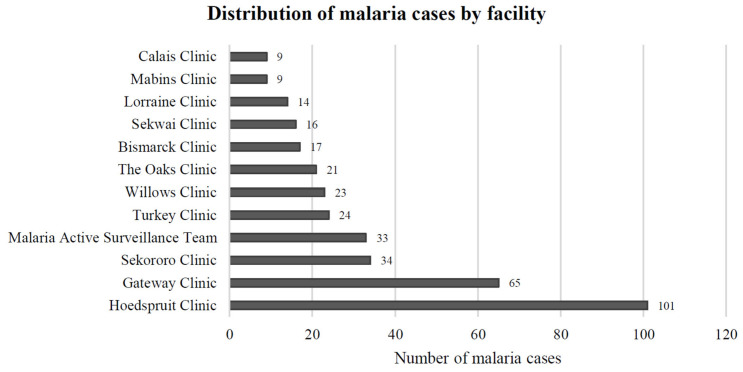
Number of malaria-positive cases reported by public healthcare facility in Maruleng Sub-District, 2018–2023. Facilities are ranked by total reported cases.

Figure 1 shows the distribution of confirmed malaria cases reported across primary healthcare (PHC) facilities in Maruleng Sub-District during the study period. The number of cases varied considerably across facilities. Hoedspruit Clinic recorded the highest number of confirmed malaria cases (*n* = 101), followed by Gateway Clinic (*n* = 65) and the Malaria Active Surveillance Team (*n* = 33). Moderate numbers of cases were reported at Sekororo Clinic (*n* = 34), Turkey Clinic (*n* = 24), Willows Clinic (*n* = 23), and The Oaks Clinic (*n* = 21). Fewer cases were recorded at Bismark Clinic (*n* = 17), Sekwai Clinic (*n* = 16), Lorraine Clinic (*n* = 14), and both Mabins Clinic and Calais Clinic (*n* = 9 each).

### 3.3. Annual Distribution of Malaria Cases

**Figure 2:**
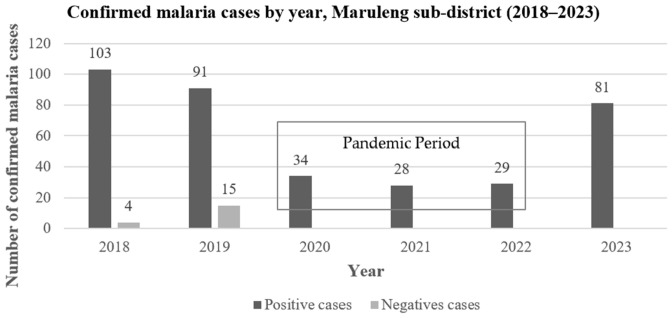
Annual number of malaria-positive and malaria-negative test results among adults presenting to public health facilities in Maruleng Sub-District, Limpopo Province, South Africa, 2018–2023. Annual counts should be interpreted descriptively because annual testing denominators were unavailable.

Malaria cases were highest in 2018 (*n* = 103) and 2019 (*n* = 91), followed by a marked decline between 2020 and 2022, with 34, 28, and 29 cases reported, respectively (Figure 2). An increase in cases was observed again in 2023 (*n* = 81). Only a small number of negative malaria test results were recorded throughout the study period, indicating that testing was primarily conducted among clinically suspected malaria cases only. Annual counts were interpreted descriptively because annual testing denominators were unavailable.

### 3.4. Seasonal Distribution of Malaria Cases

**Figure 3:**
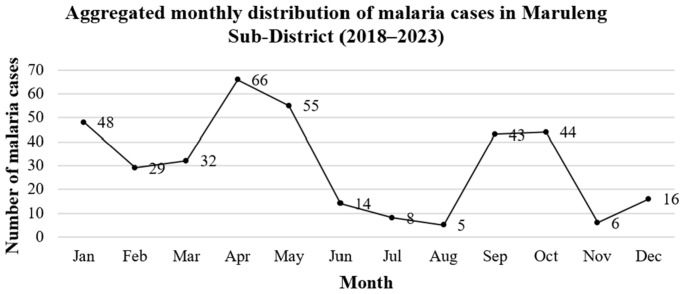
Monthly distribution of confirmed malaria cases among adults in Maruleng Sub-District, Limpopo Province, South Africa, aggregated across the study period, 2018–2023 (*n* = 366).

Figure 3 presents the monthly distribution of confirmed malaria cases aggregated across the study period (2018–2023). Malaria cases occurred throughout the year but demonstrated marked seasonal variation, peaking in January (*n* = 48), April (*n* = 66), May (*n* = 55), September (*n* = 43) and October (*n* = 44). The lowest numbers of cases were observed in July (*n* = 8), August (*n* = 5) and November (*n* = 6). A chi-square goodness-of-fit test indicated that reported malaria cases were not evenly distributed across months (χ^2^ = 155.70, df = 11, *p* < 0.001), suggesting the presence of seasonal variation in case occurrence.

### 3.5. Factors Associated with Malaria Positivity

Exploratory logistic regression analyses were performed to investigate factors potentially associated with malaria positivity (Table 2). Assessment of multicollinearity demonstrated no evidence of problematic correlation among the explanatory variables considered for inclusion in the regression models. All variance inflation factor (VIF) values were below 5.0, indicating that multicollinearity was unlikely to have materially influenced the regression estimates. Because only 19 malaria-negative records were available for comparison, the regression analyses were based on a highly imbalanced dataset with limited outcome variation. This increased the risk of overfitting, sparse-data bias, and unstable parameter estimates, with several variables exhibiting complete separation that prevented reliable estimation of conventional logistic regression odds ratios. Consequently, the regression findings should be regarded as exploratory rather than confirmatory and interpreted with caution. The observed associations should therefore be considered hypothesis-generating rather than definitive evidence of association.

## 4. Discussion

The findings of this study should be interpreted within the context of a facility-based surveillance population. Consequently, the observed demographic patterns and associated factors reflect characteristics of adults tested for malaria rather than risk factors for malaria infection in the general population. This retrospective study demonstrates that malaria remains an ongoing public health concern in the Maruleng area of Limpopo Province despite sustained malaria control efforts and national elimination initiatives. A high malaria test positivity rate was observed among clinically suspected adult cases between 2018 and 2023, reflecting the targeted diagnostic approach used in clinical settings where testing is largely restricted to individuals with malaria-compatible symptoms. Nevertheless, the findings indicate that malaria continues to be diagnosed among adults presenting to public healthcare facilities within the sub-district and remains an important focus of local surveillance and control efforts.

The temporal analysis revealed substantial fluctuations in malaria cases over the study period. In addition to annual variation, the study demonstrated significant seasonal variation, with confirmed malaria cases peaking between January and May and declining during the winter months. The seasonal analysis was based on aggregated monthly case counts across the study period and was intended to provide a descriptive overview of temporal patterns in reported malaria cases. Because monthly testing denominator data were unavailable, it was not possible to calculate monthly positivity rates or assess whether observed seasonal patterns were influenced by variations in testing practices over time. Furthermore, aggregating data across years may obscure interannual variation in malaria occurrence. This finding is consistent with the influence of rainfall, temperature, and humidity on mosquito breeding and malaria transmission in endemic regions [11]. The highest numbers of cases were recorded in 2018 and 2019, followed by a pronounced decline between 2020 and 2022, and a subsequent increase in 2023. The decline observed during the 2020–2022 period coincided with the COVID-19 pandemic and associated public health restrictions. Reduced population mobility, including limitations on cross-border travel and local movement [14]. represents one possible explanation for the reduction in reported malaria cases. However, the present study did not assess mobility patterns, healthcare utilisation, testing practices, surveillance activities, reporting processes, or resource allocation during this period. Therefore, the observed decline may also reflect changes in healthcare-seeking behaviour, malaria testing, surveillance operations, data recording, malaria control activities, or true changes in malaria occurrence. The relative contribution of these factors could not be determined from the available data. Similar temporal patterns have been documented in malaria-endemic regions where human mobility strongly influences transmission dynamics [14,15,16].

Cross-border transmission remains a key challenge for malaria elimination in South Africa. Although the majority of cases in this study occurred among South African nationals, a proportion of infections were associated with individuals originating from neighbouring malaria-endemic countries such as Mozambique and Zimbabwe. Previous studies have demonstrated that imported malaria cases continue to contribute to ongoing transmission in South Africa, particularly in border provinces such as Limpopo [11]. Migrant labour associated with agricultural production, tourism, and hospitality industries may increase exposure to mosquito vectors and facilitate cross-border transmission [9]. These findings reinforce the need for strengthened regional collaboration and coordinated malaria control strategies across southern African countries.

The demographic distribution of malaria cases observed in this study is consistent with patterns reported in other malaria-endemic regions. Males accounted for a substantially higher proportion of cases than females, and most infections occurred among younger adults aged 18–35 years. This pattern has been widely reported across sub-Saharan Africa, where working-age males often experience greater occupational and behavioural exposure to mosquito vectors [12,17]. Outdoor occupational activities, evening social interactions, and agricultural or tourism-related work environments may increase exposure to mosquito bites during peak vector activity periods. Similar findings have been reported in studies conducted in Ethiopia [18,19], Ghana [20,21], and Tanzania [21], where malaria infection was also found to be more common among economically active adult males due to occupational exposure and behavioural risk factors. The behavioural risk factor variable available in the surveillance dataset represented a routine operational classification and did not capture specific behaviours associated with malaria exposure. Therefore, comparisons with studies examining detailed behavioural determinants should be interpreted cautiously.

The exploratory regression analysis provided preliminary insight into factors that may be associated with malaria positivity within the surveillance population. Because only 19 malaria-negative records were available for comparison, the regression analyses were based on a highly imbalanced dataset with limited outcome variation. This increased the risk of overfitting, sparse-data bias, and unstable parameter estimates, with several variables exhibiting complete separation that prevented reliable estimation of conventional logistic regression odds ratios. As such, the regression findings should be regarded as exploratory rather than confirmatory and interpreted with caution. The observed associations should therefore be considered hypothesis-generating rather than definitive evidence of association. Interestingly, the composite socioeconomic risk factor variable was associated with lower odds of malaria positivity in the univariate analysis. This finding was unexpected given that the variable included indicators commonly associated with increased malaria vulnerability, including agricultural labour, poverty, unemployment, inadequate housing, and low educational attainment. The association was attenuated and no longer statistically significant after adjustment for potential confounders. Several explanations are possible, including misclassification within routine surveillance data, residual confounding, differential healthcare utilisation, or instability arising from the small number of malaria-negative records available for comparison. Consequently, this finding should be interpreted cautiously and should not be considered evidence that socioeconomic disadvantage is protective against malaria infection. A considerable proportion of malaria cases occurred among unemployed individuals, reflecting broader socioeconomic inequalities that may influence exposure and vulnerability. Poverty, inadequate housing, limited access to healthcare services, and poor environmental sanitation are well-recognised determinants of malaria transmission in endemic settings [7,22]. Socioeconomic conditions remain important contextual determinants of malaria vulnerability in Maruleng Sub-District. However, because the surveillance database captured socioeconomic risk as a routine composite Yes/No classification rather than individual measures of income, housing quality, education, or access to healthcare, the influence of specific socioeconomic determinants could not be evaluated directly in the present study.

Environmental factors also likely contribute to the persistence of malaria transmission in the study area. Maruleng is characterised by rural settlements, irrigation farming, dams, and proximity to wildlife reserves and conservation areas. These ecological features create favourable breeding environments for mosquito vectors. Climatic conditions such as rainfall, temperature, and humidity further influence mosquito population dynamics and malaria transmission patterns. Previous research has demonstrated that living near stagnant water sources significantly increases the likelihood of malaria infection due to increased mosquito breeding habitats [7]. Environmental factors such as irrigation practices, vegetation cover, and poor drainage may contribute to malaria transmission in the sub-district. However, these factors were not directly measured, and their influence could not be assessed in the present study.

Spatial differences in case distribution were observed across health facilities within the sub-district. Hoedspruit Clinic recorded the highest number of confirmed malaria cases, followed by Gateway Clinic and the Malaria Active Surveillance Team. However, these findings should be interpreted cautiously, as facility-level case counts may be influenced by differences in catchment populations, healthcare-seeking behaviour, referral patterns, clinic size, and surveillance activities. Because denominator data were unavailable, the findings do not permit identification of malaria transmission hotspots but rather indicate facilities reporting higher numbers of malaria cases during the study period. These facilities may warrant enhanced surveillance, targeted vector control activities, and further investigation using denominator-based and geospatial data.

The findings of this study have important implications for malaria elimination policy in South Africa. The country has committed to malaria elimination as part of the WHO Global Technical Strategy for Malaria 2016–2030 and its national malaria elimination strategic plan. Despite substantial progress in reducing malaria transmission over the past decades, the persistence of malaria cases in sub-districts such as Maruleng illustrates ongoing challenges in achieving elimination targets. Environmental conditions, socioeconomic vulnerabilities, and cross-border transmission remain important barriers to malaria elimination [23]. Strengthening surveillance, enhancing cross-border collaboration, and targeting interventions in high-risk areas will be critical to achieving national elimination goals. The findings also highlight the importance of robust local surveillance and early detection systems. Routine surveillance data can provide valuable insights into malaria trends and transmission patterns, helping to identify high-risk areas and guide control efforts Integrating surveillance data with environmental, climate, and population mobility information could further improve the timely detection of emerging high-risk areas and support more effective, data-driven malaria control and elimination strategies.

This study adds important sub-district-level evidence on malaria epidemiology in Limpopo Province, addressing a critical gap in the local literature. Understanding where, when, and among whom malaria occurs is essential for targeting interventions, optimising resources, and accelerating progress towards elimination. Sustained surveillance, integrated vector control, and strengthened cross-border collaboration will be key to reducing transmission and achieving South Africa’s malaria elimination goals.

### 4.1. Strengths

This study provides valuable sub-district-level insights into malaria epidemiology using six years (2018–2023) of routine surveillance data. The inclusion of records from multiple public health facilities enabled the assessment of annual and seasonal malaria trends among adults seeking care in Maruleng Sub-District. The use of confirmed malaria diagnoses improved the reliability of case classification, while the inclusion of demographic, behavioural, and socioeconomic variables facilitated exploration of factors associated with malaria positivity. The study provides locally relevant epidemiological evidence to support malaria surveillance, seasonal preparedness, and elimination planning at sub-district level.

### 4.2. Limitations

Despite the study’s strengths, several limitations should be considered. First, the study relied on retrospective routine surveillance data, which may be affected by missing, incomplete, or inconsistently recorded information. In addition, the socioeconomic and behavioural variables were limited to routine surveillance classifications and did not provide detailed information on the severity or nature of specific risk factors, such as income level, educational attainment, housing conditions, mobility patterns, or preventive practices. Consequently, the influence of these determinants on malaria risk could not be comprehensively assessed. Second, the study included only individuals who attended public health facilities presenting with clinical symptoms of malaria and were tested for malaria. Furthermore, annual analyses were based on reported malaria case counts rather than incidence rates. Because annual testing volumes and population denominator data were unavailable, changes in case numbers over time may reflect variations in testing, healthcare utilisation, surveillance activities, or reporting practices rather than true changes in malaria occurrence. Therefore, the findings cannot be used to estimate population-level malaria risk, prevalence, or incidence in the broader Maruleng population. Facilities reporting higher case numbers, such as Hoedspruit and Gateway, should not be classified as hotspots because catchment population denominators and geospatial transmission data were unavailable. Third, seasonal patterns were assessed using monthly case frequencies, but environmental and climatic factors such as rainfall, temperature, and humidity were not available for analysis. Consequently, the drivers of the observed seasonal variation could not be directly examined. Fourth, only 19 malaria-negative records were available for comparison, resulting in a highly imbalanced dataset for the regression analyses. This limited statistical power and may have increased the risk of overfitting, sparse-data bias, and unstable parameter estimates, as reflected by the wide confidence intervals observed for several variables. Consequently, the regression findings should be regarded as exploratory rather than confirmatory and interpreted with considerable caution. Future studies with larger numbers of malaria-negative records should consider penalised or exact logistic regression methods for sparse datasets. Additionally, the study utilised routine surveillance data from public healthcare facilities, which serve the majority of the South African population. Nevertheless, malaria cases diagnosed and managed exclusively within the private healthcare sector were not captured. This may be particularly relevant in Maruleng Sub-District, where tourism-related activities and visitors may contribute to greater utilisation of private healthcare services. Consequently, some malaria cases may not have been captured by the surveillance system, potentially affecting the representativeness of the study population and limiting the generalisability of the findings. Lastly, the observational design precludes causal inference. Future prospective, population-based studies incorporating environmental and climate data are needed to better understand malaria transmission dynamics in the region.

## 5. Conclusions

In conclusion, malaria remains an important public health concern in the Maruleng Sub-District of Limpopo Province. Malaria cases showed marked temporal and seasonal variation, with transmission occurring throughout the year but peaking between January and May. The higher positivity among suspected young adult males suggests that occupational and behavioural factors may contribute to risk, while higher numbers of reported cases at selected facilities may indicate areas requiring enhanced surveillance and targeted malaria control activities. The positivity rate reflects a targeted symptomatic population and should not be interpreted as a measure of community-level malaria prevalence. Nonetheless, these findings highlight the need for strengthened surveillance, targeted vector control, and enhanced cross-border collaboration to support South Africa’s malaria elimination efforts. Sustained investment in malaria prevention, surveillance, and locally targeted interventions should remain a priority in Maruleng Sub-District.

### Recommendations

Based on the study findings, the following recommendations are proposed to strengthen malaria control and support elimination efforts in Maruleng Sub-District:

- Strengthen surveillance and data monitoring systems to improve the early detection of cases and rapid outbreak response. Future studies should integrate environmental and climate data to better understand transmission patterns and support early warning systems.
- Prioritise vector control interventions, including indoor residual spraying and environmental management, in identified transmission areas, particularly around facilities reporting higher case numbers.
- Enhance cross-border collaboration with neighbouring malaria-endemic countries, particularly Mozambique and Zimbabwe, through improved surveillance, information sharing, and coordinated control programmes to reduce imported infections.
- Strengthen public health education programmes that promote preventive behaviours, early healthcare seeking, and personal protection measures, with targeted messaging for high-risk groups such as working-age males and outdoor workers.
- Conduct larger, prospective studies incorporating environmental, climatic, mobility, and geospatial data to better understand transmission dynamics, identify high local transmission areas, and inform targeted interventions.

## Figures and Tables

**Table 1:** Sociodemographic and clinical characteristics of adults tested for malaria in Maruleng Sub-District, 2018–2023.

Variable	Total Cases (*n* = 385)	Malaria Cases		*p*-Value
		Negative (*n* = 19)	Positive (*n* = 366)	
**Age**, mean (SD)	38.1 (14.1)	34.2 (11.4)	38.3 (14.2)	0.220
**Age group** (years)				
18–35	186 (48.3)	12 (63.2)	174 (47.5)	0.314
36–45	89 (23.1)	2 (10.5)	87 (23.8)	
≥46	110 (28.6)	5 (26.3)	105 (28.7)	
**Sex**				
Female	126 (32.7)	4 (21.1)	122 (33.3)	0.266
Male	259 (67.3)	15 (78.9)	244 (66.7)	
**Race**				
Black	361 (93.8)	19 (100.0)	342 (93.4)	0.249
White	24 (6.2)	0 (0.0)	24 (6.6)	
**Traced**				
No	42 (10.9)	3 (15.8)	39 (10.7)	0.484
Yes	343 (89.1)	16 (84.2)	327 (89.3)	
**Nationality**				
South African	332 (86.2)	12 (63.2)	320 (87.4)	**0.003**
Non-South African	53 (13.8)	7 (36.8)	46 (12.6)	
**Employment status**				
Employed	161 (41.8)	13 (68.4)	148 (40.4)	**0.050**
Pensioner	26 (6.8)	0 (0.0)	26 (7.1)	
Scholar	4 (1.0)	0 (0.0)	4 (1.1)	
Unemployed	189 (49.1)	5 (26.3)	184 (50.3)	
Unknown	5 (1.3)	1 (5.3)	4 (1.1)	
**Outcome**				
Alive	369 (95.8)	18 (94.7)	351 (95.9)	0.804
Deceased	16 (4.2)	1 (5.3)	15 (4.1)	
**Socioeconomic risk factor**				
No	233 (60.5)	7 (36.8)	226 (61.7)	**0.030**
Yes	152 (39.5)	12 (63.2)	140 (38.3)	
**Behavioural risk factor**				
No	252 (65.5)	10 (52.6)	242 (66.1)	0.228
Yes	133 (34.5)	9 (47.4)	124 (33.9)	
**Travel history**				
No	300 (77.9)	11 (57.9)	289 (79.0)	**0.031**
Yes	85 (22.1)	8 (42.1)	77 (21.0)	

Values are presented as *n* (%) unless otherwise indicated. Percentages are column percentages. *p*-values were obtained using Pearson’s χ^2^ tests or Fisher’s exact tests, as appropriate. “Deceased” refers to individuals recorded as deceased in the surveillance database. SD = standard deviation. *p*-values < 0.05 were considered statistically significant.

**Table 2:** Exploratory crude and multivariable logistic regression analysis of factors associated with malaria positivity among suspected adult cases tested for malaria in Maruleng Sub-District, 2018–2023.

Variable	Crude OR (95% CI)	*p*-Value	Adjusted OR (95% CI)	*p*-Value
Age group (years)				
18–35	Reference		Reference	
36–45	0.76 (0.24–2.38)	0.762	0.77 (0.24–2.45)	0.655
≥46	0.78 (0.26–2.31)	0.778	0.84 (0.28–2.52)	0.754
Sex				
Female	Reference		Reference	
Male	0.95 (0.35–2.55)	1	0.87 (0.32–2.39)	0.794
Previous malaria infection				
No	Reference		NE	
Yes	NE	1	NE	
Malaria cases traced				
No	Reference		Reference	
Yes	0.38 (0.05–2.89)	0.489	0.39 (0.05–3.06)	0.371
Nationality				
Non-South African	Reference		NE	
South African	NE	0.09	NE	
Socioeconomic risk factor				
No	Reference		Reference	
Yes	1.41 (0.52–3.79)	0.632	0.78 (0.24–2.54)	0.686
Behavioural risk factor				
No	Reference		Reference	
Yes	2.87 (0.82–10.04)	0.134	3.42 (0.78–14.98)	0.102
Travel history				
No	Reference		NE	
Yes	NE	0.018	NE	

Note: Clinical outcome (alive vs deceased) was excluded from the final regression model because it occurs after malaria diagnosis and is therefore not a determinant of malaria positivity. NE = not estimable using conventional logistic regression because of zero cells/complete separation. The full multivariable model was not estimable due to sparse data and separation. Adjusted ORs are shown only for estimable covariates and should be interpreted as exploratory. Multicollinearity diagnostics indicated acceptable VIF values < 5.0 for all explanatory variables included in the multivariable model. OR = odds ratio; aOR = adjusted odds ratio; CI = confidence interval.

## Data Availability

The data supporting the findings of this study are not publicly available due to privacy and ethical restrictions imposed by the Limpopo Department of Health. Data may be available from the corresponding author upon reasonable request and with permission from the Limpopo Department of Health.

## Supplementary Materials

The following supporting information can be downloaded at: https://www.mdpi.com/article/10.3390/ijerph23070866/s1, Table S1: Data extraction sheet.

## Abbreviations

The following abbreviations are used in this manuscript:

aORs	adjusted Odd Ratios
CDC	Centers for Disease Control and Prevention
CIs	Confidence Intervals
COVID-19	Coronavirus disease 2019
LMIC	Low Middle Income Country
MMCC	Maruleng Malaria Control Centre
ORs	Odd Ratios
PHC	Primary Healthcare
SD	Standard Deviation
SPSS	Statistical Package for the Social Sciences
VBD	Vector-borne diseases
VIF	Variance Inflation Factor
WHO	World Health Organization
