# Supplementary material for: Malaria Test Positivity, Temporal Trends, and Associated Factors Among Clinically Suspected Adult Cases in Maruleng Sub-District, Limpopo Province, South Africa, 2018–2023
Source: Int J Environ Res Public Health. 2026 Jul 2;23(7):866. doi: 10.3390/ijerph23070866 (PMC13410263; doi:10.3390/ijerph23070866)
Supplement: Supplementary file 1 [file ijerph-23-00866-s001.zip › ijerph-4383006-supplementary.pdf]

DATA EXTRACTION SHEET

Section 1: General Information							Section 2: Malaria diagnosis		Section 3: Malaria infection					Section 4: Malaria Treatment and outcomes						Section 5: Risk Factors if information is available in the record					
Cli nic Na me	Loca tion	Data Collec tion date	Sex	A	Occupa tion	Dwel ling type	Date Diagno sed	Diagno stic Metho d	Malaria classifica tion	# Previ ous infect	Last infec tion date	Coexis ting health condit ions	Sympto ms Experie nced	Treatm ent Receiv ed	Aver age reco very time	Treat ment succes s rate	Treat ment Adher ence	Foll ow- up date s	Reco very date	Environ mental	Socioeco nomic	Behavi oural	Travel History Related	Mosq uito- Prone Areas	Preven tive Measu res
